# Direct observation of realistic-temperature fuel combustion mechanisms in atomistic simulations

**DOI:** 10.1039/c6sc00498a

**Published:** 2016-05-05

**Authors:** Kristof M. Bal, Erik C. Neyts

**Affiliations:** a Department of Chemistry , University of Antwerp , Universiteitsplein 1 , 2610 Antwerp , Belgium . Email: kristof.bal@uantwerpen.be

## Abstract

Atomistic simulations can in principle provide an unbiased description of all mechanisms, intermediates, and products of complex chemical processes. However, due to the severe time scale limitation of conventional simulation techniques, unrealistically high simulation temperatures are usually applied, which are a poor approximation of most practically relevant low-temperature applications. In this work, we demonstrate the direct observation at the atomic scale of the pyrolysis and oxidation of *n*-dodecane at temperatures as low as 700 K through the use of a novel simulation technique, *collective variable-driven hyperdynamics* (CVHD). A simulated timescale of up to 39 seconds is reached. Product compositions and dominant mechanisms are found to be strongly temperature-dependent, and are consistent with experiments and kinetic models. These simulations provide a first atomic-level look at the full dynamics of the complicated fuel combustion process at industrially relevant temperatures and time scales, unattainable by conventional molecular dynamics simulations.

## Abstract 2

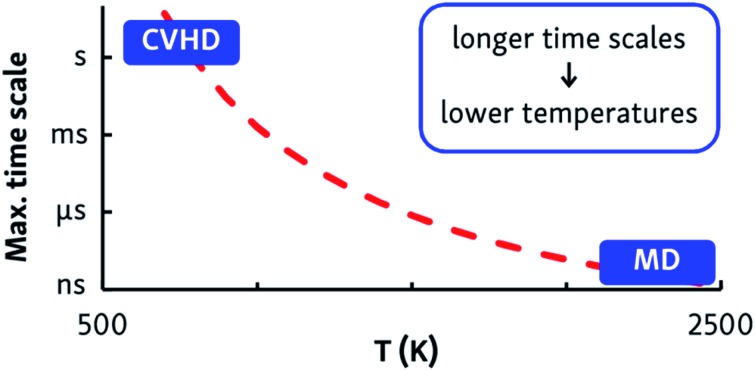
Advanced accelerated molecular dynamics simulations provide a detailed atomic-level picture of combustion at realistic temperatures and pressures.

## Introduction

A detailed understanding of pyrolysis and combustion is of great technological and industrial importance. A fundamental insight in (bio)fuel decomposition chemistry is essential to improve the selectivity of cracking and reforming processes and increase the efficiency of combustion engines and minimize their production of pollutants. For example, low temperature combustion (LTC) strategies can significantly decrease production of particulate matter (PM) and nitrogen oxides (NO_*x*_) in engines, but additional insights into their operation are required for further optimization.^1^,^2^ To screen and improve possible operating conditions, kinetic modeling can be used to explain and guide experimental investigations.^3^–^6^ It is, however, extremely challenging to create sufficiently complete and accurate kinetic models due to the wealth of possible intermediates and pathways that can all contribute significantly to the overall process, which generally limits their predictive power.

Atomistic simulation techniques can be used to bridge the gap between experimental results and kinetic models. Molecular dynamics (MD) simulations do not require any *a priori* knowledge of all possible reaction mechanisms and intermediates but generate the natural system evolution by explicitly integrating the equations of motions of all atoms. Therefore, MD simulations can be used to predict product compositions and to discover new unexpected pathways and intermediates, without any bias introduced by an incomplete reaction set. Not only can MD simulations be used to predict the outcome of a complex chemical process, but the thus obtained fundamental knowledge can also be used to extend and improve existing kinetic models. Crucial to the success of a MD simulation is the accuracy of the interatomic potential; in particular, the ReaxFF potential^7^ has been successfully applied to various pyrolysis and combustion reactions.^8^–^16^ Nevertheless, a significant limitation of MD simulations is the short (up to nanosecond) time scale they are able to reach; previous MD studies therefore invariably used very high (>2000 K) simulated temperatures to be able to observe appreciable pyrolysis or combustion within the short MD time scale. The main drawback of this approach, however, is that it is difficult to correlate insights from high-temperature simulation with industrially relevant processes at lower temperatures, such as alkane cracking at ∼1000 K or low-temperature diesel engines. In order to reach these lower operating temperatures, the simulation time scale must be drastically extended.

Applying accelerated simulation methods to fuel decomposition is extremely challenging. Methods that require saddle-point searching, such as temperature-accelerated dynamics (TAD)^17^ or on-the-fly kinetic Monte Carlo,^18^ have difficulties handling liquid- or gas-like systems, whereas force-bias Monte Carlo simulations have been successful primarily in relaxing amorphous solids.^19^ The parallel replica (ParRep) method^20^,^21^ imposes almost no constraints on the simulations and has been applied to the thermal decomposition of *n*-hexadecane^22^ and 1-hexene.^23^ In the latter case, pyrolysis could be simulated at 1350 K over a simulated time of ∼1 μs by using up to 180 replicas. A further extension of the ParRep time scale to the millisecond-to-second range necessary for capturing processes at temperatures of 1000 K or lower is, however, impractical. Indeed, because the acceleration by ParRep is proportional to the number of processors, simulating this kind of process would put unrealistic demands on available computational resources.

In principle, longer time scales can be reached with hyperdynamics, at a much smaller cost.^24^,^25^ This method operates by applying a bias potential Δ*V* to the potential energy surface, “filling” energy minima and consequently lowering the reaction activation energy. Designing a suitably general and efficient expression for Δ*V* is also the most challenging aspect of hyperdynamics. A practical problem of fuel pyrolysis and combustion simulations is the large separation of reaction barriers (and associated reaction time scales) that can be encountered during the process, ranging from ∼30 kcal mol^–1^ for alkyl radical β-scissions to ∼80 kcal mol^–1^ for initiation reactions of alkane pyrolysis. This has a major impact on the applicability of hyperdynamics, since a simple “static” bias potential can only be designed to work well for a small range of possible barriers; a bias that achieves a good acceleration or *boost factor* of β-scissions will still fall short in bringing the initiation reaction within reach. In some specific cases, a conventional hyperdynamics scheme can be sufficient: Cheng *et al.* exploited the very fast radical chemistry in hydrogen combustion, only applying a predefined bias potential to radical initiaton.^26^ However, the much longer lifetimes of hydrocarbon radicals^23^ and the employed ReaxFF-specific concepts render this approach not generally applicable.

In this work, we apply our recently proposed self-learning variant of the hyperdynamics algorithm, *collective variable-driven hyperdynamics* (CVHD) method^27^ to the initial phase of *n*-dodecane pyrolysis and combustion to, for the first time, uncover detailed atomic-level fuel decomposition pathways under realistic conditions. These simulations are the first direct atomistic simulations of fuel pyrolysis and combustion chemistry under realistic conditions and provide an additional validation of contemporary mechanistic insights.

## Computational methodology

### The CVHD method

In the CVHD method,^27^ which combines hyperdynamics with aspects of metadynamics,^28^ a suitable bias potential can be slowly “grown” during the simulation until a transition is observed. A detailed discussion of the CVHD method and a comparison with other adaptive accelerated MD methods is available in ref. 27, but here we briefly summarize its main aspects.

Crucial to the success of a CVHD simulation is the choice of an appropriate collective variable (CV) that includes the relevant degrees of freedom *s*, and their distortions from equilibrium *χ*(*s*), associated with the to-be-boosted process. CVHD uses a general functional form that is inspired by the work of Tiwary and van de Walle,^29^ and a generalization of the bond boost method.^30^ Bias and system-specific dynamics are therefore cleanly separated: any complicated dynamics is projected on a single CV *η* as a value between 0 (no distortion) and 1 (maximal distortion receiving a bias), which is the only variable on which the bias explicitly depends. Furthermore, in contrast to the bond boost method, degrees of freedom other than bond elongations can be biased. For example, the folding of a model polymer was studied with CVHD by calculating *η* from dihedral angles rather than bond lengths.^27^

As in metadynamics, a history-dependent bias potential is constructed by adding Gaussian-shaped “hills” *w* exp((*η* – *η*(*t*_i_))^2^/2*δ*^2^) at intervals *t*_i_. New hills are continuously added during the simulation to strengthen the bias, until a transition is observed. The criterion to detect a transition is time-based: if *η* remains 1 during a predefined waiting time *t*_w_, the system is assumed to have undergone a transition. Then, the bias deposition procedure is reinitiated from scratch in the new state. No bias potential is added to the system when *η* = 1, so that the correct sequence of state-to-state transitions is preserved by construction.^24^ CVHD is partially inspired by infrequent metadynamics, in which conventional metadynamics CVs are used but the bias deposition is made very slow in order to keep transition states relatively bias-free (instead of explicitly enforcing this, as is the case with CVHD's *η*).^31^,^32^ Due to the different choice of CVs and biasing parameters, the two methods do not share the same focus: infrequent metadynamics can be used to calculate highly accurate rate estimates of a specified (slow) reaction (by repeated sampling of this transition), whereas CVHD is meant to capture the natural long time scale state-to-state evolution of the full system, discovering new reaction channels on-the-fly (and not necessarily sampling any encountered reaction beyond the first pass).

The combination of a bond length-based CV and an adaptive bias potential allows CVHD to handle complicated reactive processes with a wide distribution of barriers. As a first successful application to a chemical process, the CVHD method has already been used to simulate nickel-catalyzed methane decomposition, a process of which individual steps have barriers ranging from 8 to 32 kcal mol^–1^, and time scales of several ps to ms at 800 K.^27^

### Simulation parameters

All simulations were carried out with LAMMPS^33^ and the colvars module,^34^ using the ReaxFF potential^7^ with the Chenoweth *et al.* parameter set^8^ and QEq charge equilibration,^35^ as implemented in LAMMPS.^36^ The equations of motion were integrated with a time step of 0.1 fs, and the system was initially equilibrated at the target temperature with a Langevin-type thermostat.^37^ Further sampling in the NVT ensemble was achieved through application of a Nosé–Hoover chain^38^ with a relaxation time of 0.1 ps, whereas for isotropic NPT simulations, the Martyna–Tobias–Klein (MTK) equations of motion^39^ were integrated through the scheme of Tuckerman *et al.*,^40^ using a relaxation time of 1 ps.

For pyrolysis simulations, the system consisted of 24 alkane molecules in a 50 × 50 × 50 Å^3^ periodic box, corresponding to a density of about 0.05 g cm^–3^. As local degrees of freedom we used C–C and C–H bond lengths, with as distortion function the strain *χ*_i_ = (*r*_i_ – *r*mini)/(*r*maxi – *r*mini) for every bond, as described in ref. 27. The *r*mini and *r*maxi parameters were respectively 1.55 and 2.20 Å for C–C bonds, and 1.05 and 1.65 Å for C–H bonds. The *r*maxi values were specifically chosen to be smaller than the lengths of the breaking C–C and C–H bonds in the transition states of radical β-scissions and intramolecular hydrogen atom transfers, respectively, to ensure these states remain unbiased. This choice of CV means that only events involving bond breaking are accelerated, and conformational changes are unbiased; following a similar reasoning as in previous work, low-barrier conformational dynamics can be considered to have reached equilibrium well within the time spent while waiting for a reaction.^22^ Gaussian hills of width *δ* = 0.025 and height *w* = 0.25 kcal mol^–1^ were added every *t*_i_ = 0.2 ps; the waiting time to detect events was *t*_w_ = 1 ps. CVHD simulations were carried out between 1000 and 1800 K; for comparison, unbiased MD simulations were conducted at a temperature of 2500 K.

Constant density combustion simulations were carried out for a 40 × 40 × 40 Å^3^ box containing 5 *n*-dodecane and 100 oxygen molecules, corresponding to a fuel-lean mixture with a density of about 0.1 g cm^–3^. The CVHD parameters are the same as those of the pyrolysis simulations, with all interactions involving oxygen atoms being described by the corresponding values for carbon. Biased simulations were carried out between 700 and 1800 K, and conventional MD was again performed at 2500 K. The average pressures in these simulations range from ∼200 bar at 700 K to almost 500 bar at 1800 K.

In order to capture the pressure dependence of the oxidation process over the range of pressures relevant to practical combustion applications, we also carried out a set of constant pressure simulations at 1000 K and pressures between 10 and 500 bar. A particularly important complication of CVHD simulation of gas-phase systems is that lowering the pressure also lowers the collision frequency in the system. Therefore, to prevent excessive buildup of bias between possible reactive collisions, and an overestimation of the time scale, the Gaussian deposition stride must be lowered accordingly. While 0.2 ps suffices for the high-density NVT simulations, we found that the 10 bar simulation requires a deposition interval of 0.5 ps, a value we used in all NPT simulations. When applying CVHD to other gas-phase systems, care must again be taken to choose an appropriate deposition stride.

Unless noted otherwise, all comparisons between simulations at different temperatures, such as of product compositions and time scales, are made at a fixed conversion level. For pyrolysis, analysis was performed at 50% fuel conversion. Combustion simulations were carried out until 20% of the O_2_ molecules were consumed. For every condition, two independent trajectories were calculated to obtain reliable statistics. Error intervals, if reported, reflect the 90% confidence level.

## Results and discussion

### Accessible time scale

**Fig. 1:**
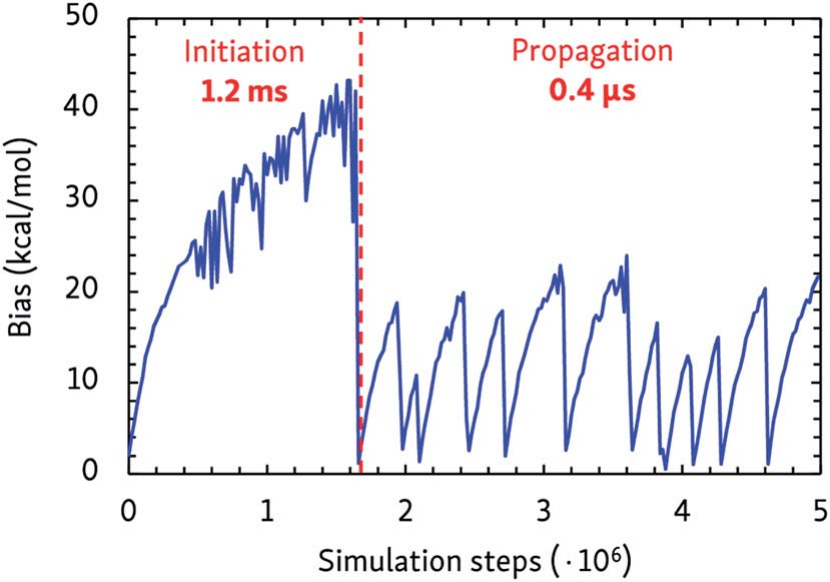
Applied maximal bias potential during the initial steps of a 1000 K CVHD pyrolysis simulation. The time scales of the two displayed distinct regimes are also shown.

The dynamic self-learning nature of the CVHD method is illustrated in Fig. 1, which shows the evolution of the applied bias potential in the first stages of a pyrolysis simulation: the bias strength is slowly increased until an event is detected, and the biasing procedure is restarted. It can also be seen that the initiation reaction, which is a C–C bond fission, is the slowest event that requires the largest bias potential, whereas subsequent radical isomerizations and β-scissions have lower barriers. Thus, the bias strength is automatically tuned to be optimal for the current stage of the simulation. As summarized in Table 1, application of CVHD allows us to observe alkane pyrolysis and combustion at temperatures as low as 1000 and 700 K, respectively; the largest boost factor in our simulations is 8 × 10^6^ larger than that of the longest pyrolysis ParRep simulation.^23^ The longest simulated physical time is therefore almost 40 s.

**Table 1:** Lowest temperatures achieved in the CVHD simulations of *n*-dodecane pyrolysis and combustion, and corresponding physical times and boost factors

	Pyrolysis	Combustion
Lowest temperature	1000 K	700 K
Longest simulated time	57 ms	39 s
Largest boost	6.3 × 10^6^	1.3 × 10^9^

### Pyrolysis

**Fig. 2:**
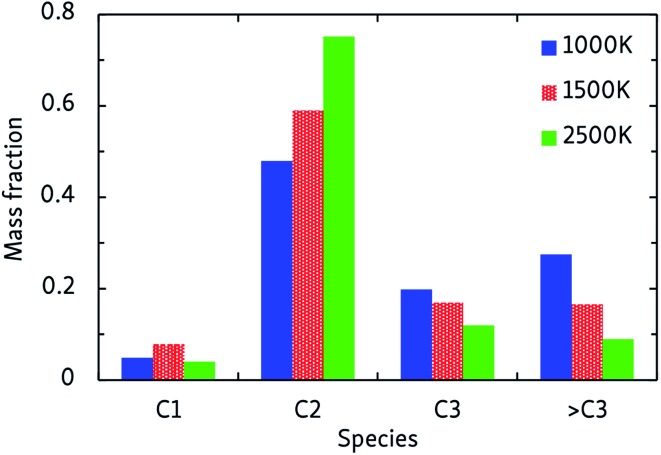
Products of the CVHD *n*-dodecane pyrolysis simulations at different temperatures.

In general, the alkane decomposition chemistry observed in the CVHD simulations is similar to previous high-temperature (>2000 K) MD simulations of alkane pyrolysis.^10^,^11^ Most reactions of large alkyl radicals are either isomerization by intramolecular H-transfer, or decomposition to 1-alkenes through β-scission (the Rice–Kossiakoff mechanism). At high temperatures, the entropically favored decomposition reactions are the dominant process: ethylene is by far the dominant reaction product, in agreement with previous high-temperature MD simulations.^10^,^11^ In contrast, low-barrier isomerization occurs much more frequently at low temperatures, forming more stable secondary radicals which give rise to the formation of larger 1-alkenes after eventually undergoing β-scission. Therefore, lower pyrolysis temperatures yield larger product molecules, as shown in Fig. 2. In contrast to the 2500 K simulation, where the C2 fraction is dominant and higher fractions are negligible, heavier molecules (C3 and higher) comprise about 50% of the products at 1000 K. Similarly, we observe that low-temperature propagation reactions involving H-abstraction by small radicals such as H, CH_3_ and C_2_H_5_ constitute the main consumption channel of unreacted alkanes, but at high temperatures unimolecular initiation through bond fission gains importance.

The relative stability of C–C and C–H bonds is also found to be temperature-dependent. Because a C–H bond is about 25 kcal mol^–1^ stronger than a C–C bond, unimolecular initiation at low temperatures only occurs through C–C dissociation; at high temperatures, considerable C–H dissociation is also observed, resulting in highly reactive free H atoms, in agreement with earlier high-temperature simulations of *n*-heptane pyrolysis.^11^ A constant supply of free H radicals has a large impact on the overall reactivity of the system and the propagation rate, again illustrating the temperature-dependence of the pyrolysis mechanism. At low temperatures, C–H dissociation is only observed in radicals: ReaxFF predicts that C–H bonds vicinal to a radical site are about 50 kcal mol^–1^ weaker than those in alkanes (dissociation energies of ∼50 and ∼100 kcal mol^–1^, respectively), significantly facilitating their dissociation. Especially the ethyl radical, which has a C–H bond dissociation energy of 45 kcal mol^–1^, frequently decomposes into C_2_H_4_ + H.

### Oxidation

**Fig. 3:**
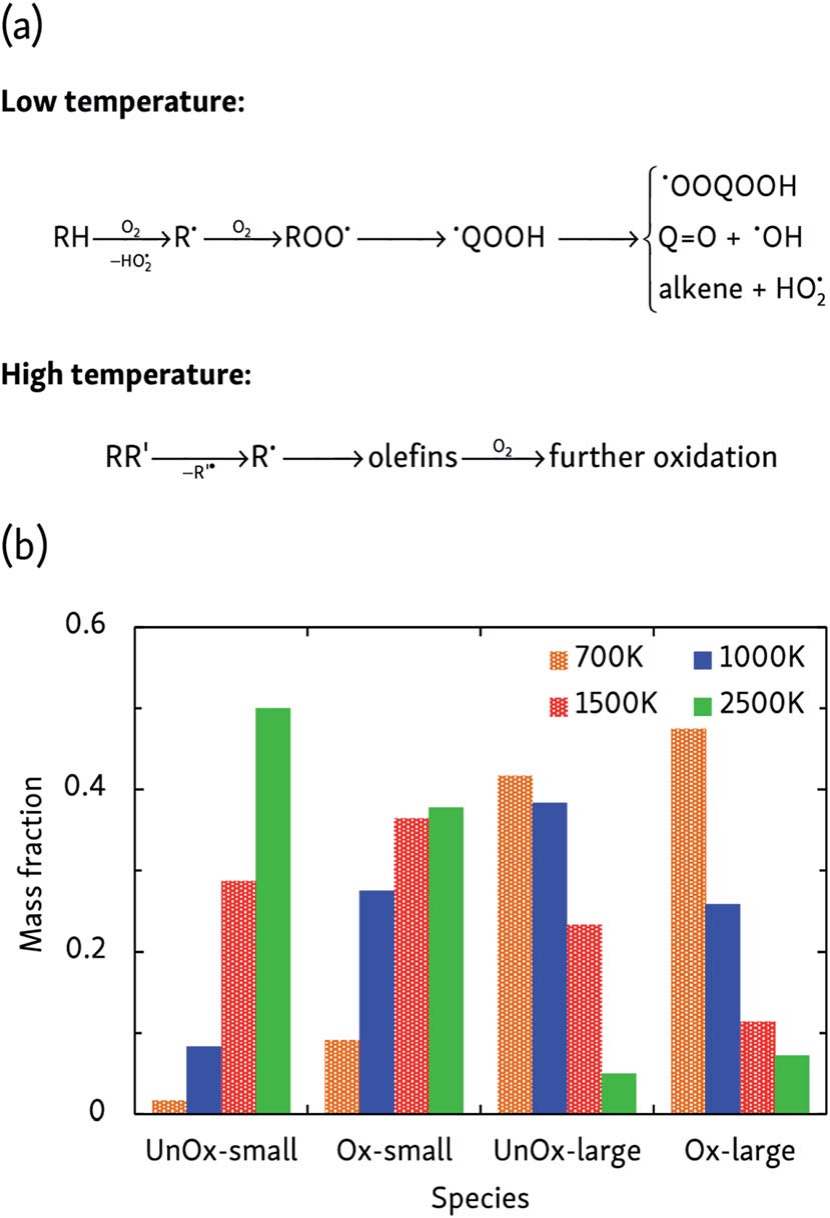
(a) Limiting temperature-dependent initial oxidation mechanisms, and (b) products of the constant-density CVHD *n*-dodecane oxidation simulations at different temperatures. *Unox* species do not contain oxygen, whereas *Ox* do; *large* products are C3 or heavier. The mass fraction is that of carbon only, and reflects how carbon is distributed over the various species.

More complicated mechanisms are observed in the oxidation simulations, of which there are two distinct limiting cases, summarized in Fig. 3a. In the low temperature mechanism, the oxidation process is always initiated by hydrogen abstraction by an oxygen molecule and the subsequently formed alkyl radical combines with another oxygen molecule to form a peroxy radical ROO˙. Further isomerization leads to a hydroperoxyalkyl radical ˙QOOH, which can react further through a variety of pathways. Additionally, further H-abstractions by O_2_ or reactive oxygen species from alkenes, radicals and carbonyl-containing compounds lead to the formation of compounds such as (conjugated) alkenes, ketenes and keto-hydroperoxides. At high temperatures, on the other hand, initial steps are essentially a pyrolysis process initiated by unimolecular C–C bond fission and subsequent β-scissions, forming primarily C_2_H_4_ which is further oxidized in a later stage.

At intermediate oxidation temperatures, both mechanisms are at play: below 1500 K, alkanes are initiated by H-abstraction but then easily break down into olefins, whereas from 1000 K and lower, C–C bond fission only rarely occurs in the initial oxidation stages. These temperature-dependent mechanisms are reflected by the product distributions of Fig. 3b. High temperatures primarily produce C_2_H_4_ and its oxidation products, whereas lowering the temperature suppresses dissociation events. In agreement with the findings of the pyrolysis simulations, alkyl radical β-scissions become less likely at lower temperatures, but the formed 1-alkenes are larger due to the relatively increased isomerization rate so that the mass fraction of produced hydrocarbons remains almost constant.

The temperature also has an impact on the formation of hydrogen peroxide and water. A first hydrogen atom transfer to O_2_ forms a hydroperoxyl radical, HO_2_, which can subsequently either abstract another hydrogen atom and form H_2_O_2_, or transfer its hydrogen atom to another radical. The further reactivity of H_2_O_2_ is strongly temperature-dependent, as it is found to be stable at low temperatures, whereas at high temperature, dissociation in two OH radicals occurs within a short time. These highly reactive OH radicals can then carry out an additional hydrogen abstraction to form H_2_O. Therefore, at low temperatures, the kinetically stable H_2_O_2_ tends to accumulate whereas high temperatures favor the formation of OH and water. Indeed, at 700 K, the H_2_O_2_ fraction accounts for ∼17% of the non-O_2_ oxygen atoms to be compared with ∼14% in the H_2_O fraction. At 1000 K, this ratio is already 20/10 and from 1200 K onwards, the H_2_O_2_ fraction is negligible while the H_2_O fraction contains about 30% of all reacted O_2_. These observations are in agreement with conceptual models of low-temperature diesel engines.^41^

**Fig. 4:**
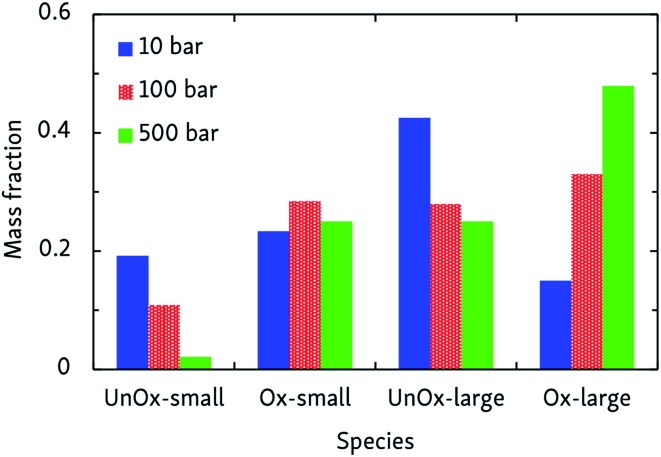
Products of CVHD *n*-dodecane oxidation simulations at different pressures, at 1000 K. Presentation of the data is the same as in Fig. 3.

Combustion chemistry is also affected by pressure, and CVHD simulations can be used to investigate this effect. If the pressure-dependent reaction rate is proportional to *p*^*n*^ and, at constant temperature and assuming ideal gas behavior, the average reaction time and, at constant temperature and assuming ideal gas behavior, the average reaction time 〈*t*〉 ∼ ∼ *p*/rate, the overall reaction order *n* can be determined by fitting ln can be determined by fitting ln〈*t*〉 = = *m* ln *p* + ln + ln〈*t*〉_*p*=1_, in which *m* = 1 – *n*. This way, we obtained *n* = 2.07 ± 0.07, indicating that the rate-determining step of the oxidation is of second order, most likely involving hydrogen abstraction. Average oxidation time scales ranged from 0.6 ms at 500 bar, to 45 ms at 10 bar. The pressure effect on the relative importance of uni- and bimolecular processes is also reflected by the product distribution, as depicted in Fig. 4. Although this effect is less pronounced than the influence temperature has on the oxidation process, it can be seen that pyrolytic mechanisms are favored at low pressures, but suppressed in denser systems.

### Comparison with experiments and unbiased MD

Our CVHD simulations also compare well with experimental results and existing kinetic models. The product distribution of the 1000 K pyrolysis process can be compared with a recent experimental study at the same temperature, in which the product distribution 0.08/0.44/0.23/0.25 of C1 through >C3 was obtained, in good agreement with our results.^42^ Moreover, the half-life of *n*-dodecane was found to be in the order of 20–40 ms, which compares well with the results in Table 1. The temperature-dependent oxidation mechanisms observed in CVHD simulations are also consistent with generally accepted models^3^,^4^ and experiments.^42^

There exists some discrepancy between our simulations and oxidation experiments. While experimentally, an early pyrolytic stage is already observed at 1050 K, our simulations suggest that this requires higher temperatures above 1200 K. This can be attributed to the high pressures in our constant density simulations, which will favor bimolecular over unimolecular reactions and thus a relative decrease of β-scissions over alkyl radical reactions with oxygen-containing species. Indeed, as shown earlier, lowering the pressure in our 1000 K CVHD simulation suppresses bimolecular reactions and gives rise to an early pyrolytic stage at lower temperatures than suggested by high-pressure simulations.

**Fig. 5:**
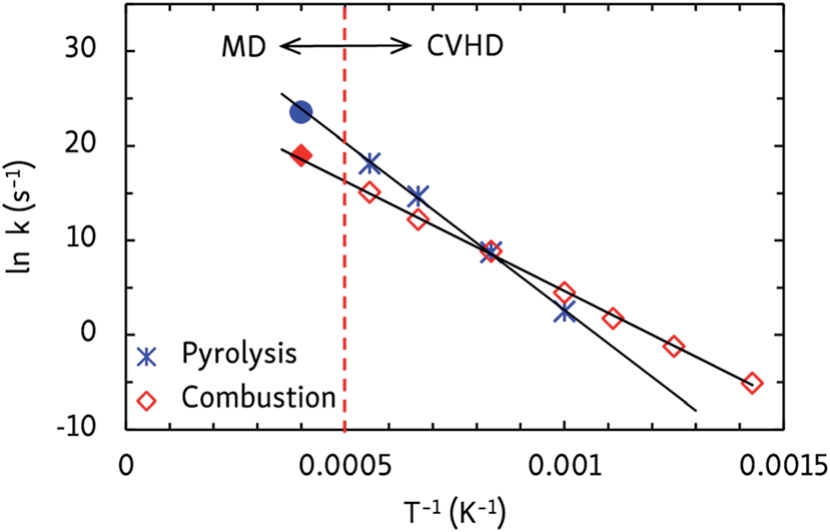
Arrhenius plots of the apparent first order rate constants of *n*-dodecane pyrolysis and combustion as obtained from CVHD simulations. Filled symbols at 2500 K are unbiased MD simulations that were not included in the fit.

Finally, apparent first order rate constants for pyrolysis and combustion were computed from the C_12_H_26_ and O_2_ consumption rates, respectively. By fitting the Arrhenius equation, prefactors *A* and activation energies *E*_A_ were obtained, and activation enthalpies Δ^‡^*H* and entropies Δ^‡^*S* were calculated from the Eyring equation, which are collected in Table 2. The pyrolysis parameters are consistent with other ReaxFF pyrolysis studies of *n*-dodecane, in which values of *E*_A_ between 56 and 66 kcal mol^–1^ and *A* from 10^15^ to 10^16^ s^–1^ are found,^10^ and with a unimolecular C–C dissociation as rate-determining step, as the positive entropy of activation indicates. For combustion, the activation energy matches that of a hydrogen abstraction by O_2_. Indeed, experimental barriers of hydrogen atom transfers from alkanes to O_2_ lie between 44 and 51 kcal mol^–1^ (ref. 43) and ReaxFF predicts a barrier of ∼50 kcal mol^–1^ for O_2_-mediated hydrogen abstraction from methane.^8^ The negative Δ^‡^*S* value for combustion is also in line with a bimolecular mechanism. This means that hydrogen atom transfers to O_2_ are rate-determining at all temperatures, regardless of the different temperature-dependent initial reaction steps. Furthermore, as can be seen from the Arrhenius plots in Fig. 5, the CVHD values are also consistent with unbiased MD simulations: extrapolation of the CVHD results to higher temperatures agree with the MD results, therefore further validating the application of CVHD to pyrolysis and combustion.

**Table 2:** Kinetic parameters of *n*-dodecane pyrolysis and combustion as obtained from fitting apparent first order Arrhenius and Eyring equations

	Pyrolysis	Combustion
Temperature range (K)	1000–1800	700–1800
*E*_A_ (kcal mol^–1^)	70 ± 5	46 ± 1
*A* (s^–1^)	5 × 10^15^ to 2 × 10^17^	7 × 10^11^ to 3 × 10^12^
Δ^‡^*H* (kcal mol^–1^)	68 ± 5	44 ± 1
Δ^‡^*S* (cal mol^–1^ K^–1^)	12 ± 4	–8 ± 1

## Conclusions

We have applied a recently developed self-learning hyperdynamics implementation, the CVHD method, to pyrolysis and combustion of the *n*-dodecane model fuel. Owing to the unprecedented long time scale of our simulations, we were able to conduct the first explicit verification of temperature- and pressure-dependent pyrolysis and combustion mechanisms through direct atomistic simulations. Reaction pathways uncovered by CVHD simulations agree well with experiments and kinetic models and suggests CVHD's ability to extend and supplement chemical kinetic models. Moreover, these results show that a flexible accelerated molecular dynamics method such as CVHD can give access to the long timescale dynamics of complex chemical processes, and how it can further extend the interpretive and predictive power of atomistic simulations by bridging the gap between theory and experiment.
